# Characteristics of Mental Health Specialists Who Shifted Their Practice Entirely to Telemedicine

**DOI:** 10.1001/jamahealthforum.2023.4982

**Published:** 2024-01-26

**Authors:** Ruth Hailu, Haiden A. Huskamp, Alisa B. Busch, Lori Uscher-Pines, Pushpa Raja, Ateev Mehrotra

**Affiliations:** 1 Department of Health Care Policy, Harvard Medical School, Boston, Massachusetts; 2 Division of General Internal Medicine, Beth Israel Deaconess Medical Center, Boston, Massachusetts; 3 McLean Hospital, Belmont, Massachusetts; 4 RAND Corporation, Arlington, Virginia; 5 Greater Los Angeles VA Medical Center, Los Angeles, California

## Abstract

This cohort study investigates the number and characteristics of US mental health specialists who had shifted to a fully virtual practice as of 2022.

## Introduction

The COVID-19 pandemic–related shift to telemedicine has been particularly prominent and sustained in mental health care. In 2021, more than one-third of mental health visits were conducted via telemedicine.^1^ While most mental health specialists have in-person and telemedicine visits, some have transitioned to fully virtual practice, perhaps for greater work-life flexibility (including avoiding commuting) and eliminating expenses of maintaining a physical clinic. The decision by some clinicians to practice only via telemedicine has gained importance due to Medicare’s upcoming requirement, effective in 2025, that patients have an annual in-person visit to receive telemedicine visits for mental illness and new requirements from some state Medicaid programs that clinicians offer in-person visits.^2^ We assessed the number and characteristics of mental health specialists who have shifted fully to telemedicine.

## Methods

This cohort study used national, deidentified commercial health insurance claims from OptumLabs Data Warehouse for commercial insurance and Medicare Advantage enrollees from January 1 to December 31, 2019, and January 1 to December 31, 2022. Harvard Medical School exempted this study from review and informed consent because data were deidentified. We followed the STROBE guideline.

We identified mental health specialists (psychiatrists, psychologists, social workers, and psychiatric mental health nurse practitioners [PMHNPs])^3^ who had at least 30 visits and 5 patients in both 2019 and 2022 and conducted less than 25% of visits virtually in 2019 (eTable in Supplement 1). Clinicians defined as “telemedicine only” conducted more than 95% of visits virtually in 2022. We did not use 100% because of potential billing errors. For each clinician, we captured specialty, sex, US region, whether most patients were younger than 18 or older than 65 years, proportion of patients with severe mental illness (schizophrenia or bipolar disorder), and median house value and population per square mile in the county where most of their patients resided (eAppendix in Supplement 1). We ran a multivariable logit model in SAS, version 9.4, on the likelihood a clinician provided telemedicine-only care in 2022 by clinician variables and present marginal effect estimates. Two-sided *P* < .05 was significant.

## Results

Among 51 309 mental health specialists meeting our inclusion criteria, 13.0% provided telemedicine-only care in 2022 (Figure). The adjusted rate was highest among PMHNPs (18.7%; 95% CI, 17.1%-20.3%) and lowest among psychiatrists (9.1%; 95% CI, 8.6%-9.7%). In multivariable models, characteristics associated with greater likelihood of switching to telemedicine only were being female (adjusted rate, 14.0% [95% CI, 13.6%-14.3%] vs 11.1% [95% CI, 10.6%-11.6%] for males; *P* < .001) and working in counties in the top (vs lowest) quartile of housing value (16.6% [95% CI, 15.9%-17.4%] vs 8.8% [95% CI, 8.2%-9.4%]; *P* < .001) and population density (16.0% [95% CI, 15.4%-16.7%] vs 8.8% [95% CI, 8.3%-9.4%]; *P* < .001) (Table). Clinicians with a pediatric focus were less likely than general clinicians to have a telemedicine-only practice (6.7% [95% CI, 6.0%-7.5%] vs 14.1% [95% CI, 13.8%-14.4%]; *P* < .001).

**Figure.**
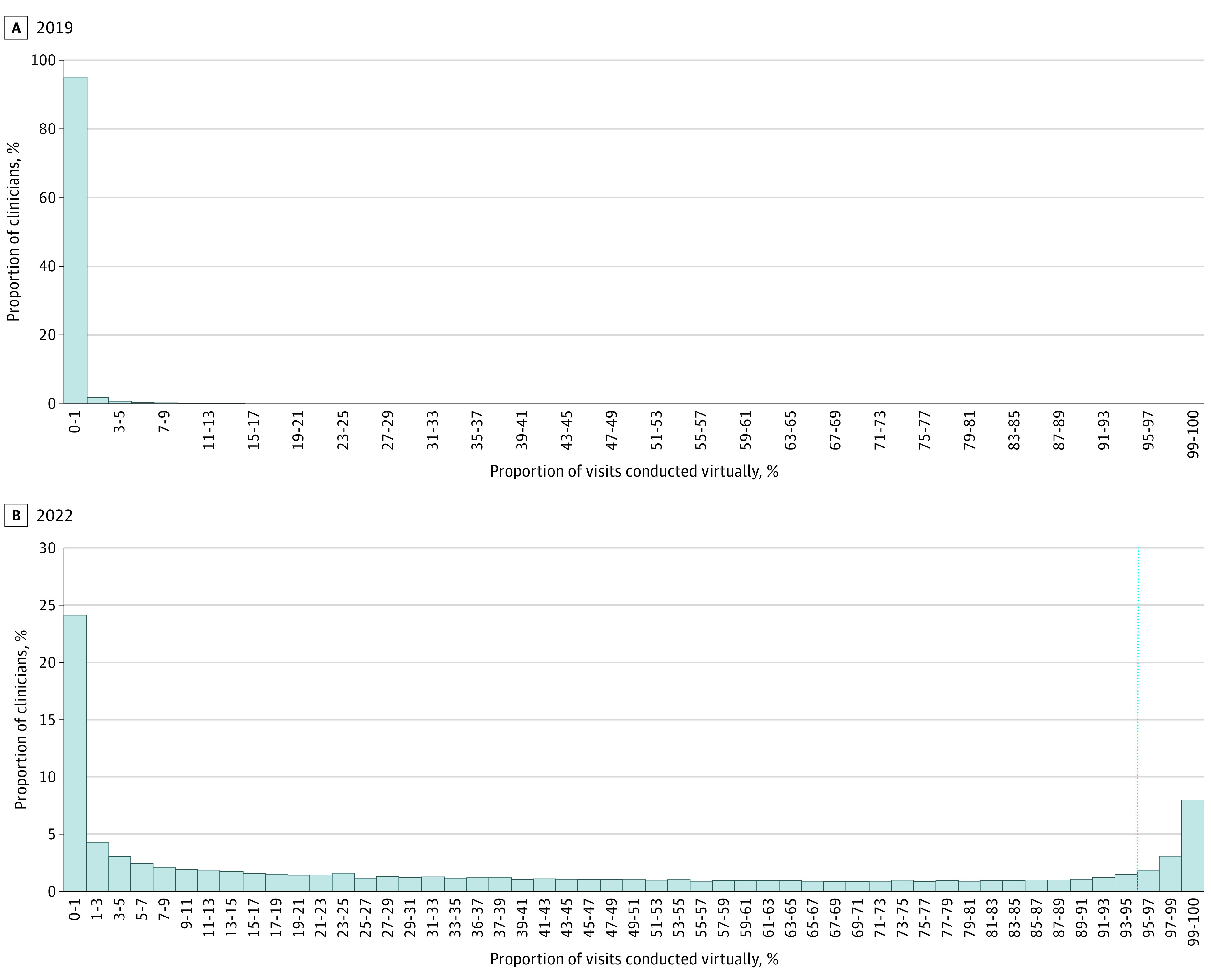
Distribution of Clinicians by Percentage of Virtual Visits in 2019 vs 2022 B, Vertical dotted line indicates the virtual-only cutoff point.

**Table.** Mental Health Clinician and Practice Characteristics Associated With Having a Telemedicine-Only Practice in 2022

Characteristic	Clinicians, No. (%)		Adjusted rate of telemedicine only, % (95% CI)^a^	*P* value
	Total (N = 51 309)	Telemedicine only (n = 6682)		
Specialty				
Psychiatrist	12 472 (24.3)	1178 (9.5)	9.1 (8.6-9.7)	NA
Psychologist	9594 (18.7)	1260 (13.1)	12.8 (12.2-13.5)	<.001
Social worker	27 115 (52.8)	3826 (14.1)	14.5 (14.1-14.9)	<.001
PMHNP	2128 (4.1)	418 (19.6)	18.7 (17.1-20.3)	<.001
Sex				
Female	33 744 (65.8)	4844 (14.4)	14.0 (13.6-14.3)	<.001
Male	17 565 (34.2)	1838 (10.5)	11.1 (10.6-11.6)	NA
US region				
Northeast	10 388 (20.2)	1839 (17.7)	14.0 (13.3-14.7)	NA
Midwest	17 631 (34.4)	1474 (8.4)	9.0 (8.5-9.5)	<.001
South	15 861 (30.9)	2087 (13.2)	15.0 (14.4-15.7)	<.001
West	7429 (14.5)	1282 (17.3)	16.3 (15.4-17.2)	<.001
Median house value in county, quartile^b^				
1	12 832 (25.0)	948 (7.4)	8.8 (8.2-9.4)	NA
2	12 810 (25.0)	1378 (10.8)	11.6 (11.0-12.2)	<.001
3	12 788 (24.9)	1834 (14.3)	13.7 (13.1-14.3)	<.001
4	12 879 (24.9)	2522 (19.6)	16.6 (15.9-17.4)	<.001
Population per square mile, quartile^c^				
1	12 833 (25.0)	978 (7.6)	8.8 (8.3-9.4)	NA
2	12 813 (25.0)	1578 (12.3)	11.9 (11.4-12.5)	<.001
3	12 798 (24.9)	2056 (16.1)	14.7 (14.1-15.3)	<.001
4	12 865 (25.1)	2070 (16.1)	16.0 (15.4-16.7)	<.001
Age of patient population				
General	43 768 (85.3)	6202 (14.2)	14.1 (13.8-14.4)	NA
Pediatric focus	4821 (9.4)	294 (6.1)	6.7 (6.0-7.5)	<.001
Older adult focus	2720 (5.3)	186 (6.8)	6.5 (5.6-7.4)	<.001
Visits with schizophrenia or bipolar diagnosis code, %				
0	24 755 (48.2)	3337 (13.5)	12.6 (12.1-13.0)	NA
0.9-7.3	8842 (17.2)	1192 (13.5)	13.4 (12.7-14.1)	.02
7.3-20.3	8859 (17.3)	1267 (14.3)	15.0 (14.3-15.8)	<.001
20.3-100.0	8853 (17.3)	886 (10.0)	11.9 (11.2-12.6)	.16

Abbreviations: NA, not applicable (reference category); PMHNP, psychiatric mental health nurse practitioner.

^a^ Marginal effect estimates and *P* values are from a multivariable logistic regression model adjusting for specialty, sex, region, quartile of median house value, quartile of population per square mile, and characteristics of the clinician’s patient population.

^b^ Quartile 1 is from $23 333 to $69 067; 2, $69 167 to $89 833; 3, $89 933 to $133 667; and 4, $113 833 to $521 234.

^c^ Quartile 1 is from 1.9 to 429.3; 2, 429.4 to 1151.8; 3, 1153.2 to 2246.9; and 4, 2249.0 to 70 608.4.

## Discussion

In 2022, 13.0% of mental health specialists serving commercially insured or Medicare Advantage enrollees had shifted to telemedicine only. Rates were higher among female clinicians and those working in densely populated counties with higher real estate prices. A virtual-only practice allowing clinicians to work from home may be more attractive to female clinicians, who report spending more time on familial responsibilities,^4^ and those facing long commutes and higher office-space costs.

It is unclear how telemedicine-only clinicians will navigate new Medicare and Medicaid requirements for in-person care. While clinicians and patients may prefer in-person care,^5^ introducing in-person requirements for visits and prescribing could cause care interruptions, particularly for conditions such as opioid use disorder.^6^

Our analysis is limited to clinicians treating patients with commercial insurance or Medicare Advantage and therefore may lack generalizability. We were also unable to determine where clinicians physically practiced, particularly if they had transitioned to virtual-health companies. Given the shortage of mental health clinicians, future research should explore whether a virtual-only model affects clinician burnout or workforce retention.

## Notes

Supplement 1.
eAppendix
**eTable.** Distribution of Visits in 2019 by Diagnostic Category Among Telemedicine-Only Clinicians vs Other Clinicians

Supplement 2.
Data Sharing Statement
