# Supplementary material for: Characteristics of Mental Health Specialists Who Shifted Their Practice Entirely to Telemedicine
Source: JAMA Health Forum. 2024 Jan 26;5(1):e234982. doi: 10.1001/jamahealthforum.2023.4982 (PMC10818220; doi:10.1001/jamahealthforum.2023.4982)
Supplement: Supplement 1. — eAppendix eTable. Distribution of Visits in 2019 by Diagnostic Category Among Telemedicine-Only Clinicians vs Other Clinicians [file jamahealthforum-e234982-s001.pdf]

Supplementary Online Content

Hailu R, Huskamp HA, Busch AB, Uscher-Pines L, Raja P, Mehrotra A. Characteristics of mental health specialists who shifted their practice entirely to telemedicine. *JAMA Health Forum*. 2024;5(1):e234982. doi:10.1001/jamahealthforum.2023.4982

eAppendix
eTable. Distribution of Visits in 2019 by Diagnostic Category Among Telemedicine-Only Clinicians vs Other Clinicians

This supplementary material has been provided by the authors to give readers additional information about their work.

© 2024 Hailu R et al. *JAMA Health Forum*.

eAppendix

**Outcome of Interest**	**Definition**
Patient Age	Pediatric focused: >2/3 of visits are with patients less than 18 years old<br>Elderly focused: >2/3 of visits are with patients over 65 years old<br>General: All other clinicians
County-Level Characteristics	Clinicians were assigned to the county in which a majority of their patients in these data resided. They were then separated into four quartiles for median house value and population per square mile.
Clinician Region	Clinicians were assigned to the Census region in which a majority of their patients in these data resided.
Patient Population with Severe Mental Illness	Clinicians were separated into four groups based on the percentage of visits that had a primary diagnosis code for schizophrenia or bipolar disorder. We first create one group of clinicians with no patients with schizophrenia or bipolar and then divided the remaining clinicians into three groups with a similar number of clinicians.
Clinician specialty	There is a specialty variable provided in the dataset and we assigned clinicians to the specialty listed most frequently in the data. While we felt it was a reasonable assumption that all social workers, psychologists, and psychiatrists were mental health specialists, we did not think this was reasonable assumption for nurse practitioners (NPs). Most NPs work on other clinical areas such as primary care. That is why we used a claims-based approach to identify NPs who focus on mental health treatment. This was defined as greater than 80% of their visits had a primary or secondary mental health diagnosis code.
Median house value	We divided up counties with a clinician in our sample into four quartiles. We use median house value as a proxy for rental prices and what a clinician might be paying to pay for their clinic site.

© 2024 Hailu R et al. *JAMA Health Forum*.

Population density	We were interested in commuting time and used population density as a rough proxy for how long it might take a clinician to get to their in-person clinical site. Unfortunately, our data use agreement does not allow us to link in other data sources which might have more detailed information on commuting time. Counties were divided into four quartiles.

*ICD10 codes for mental health diagnoses:

**Dx Category**	**ICD10**
Depressive disorder	F32.xx, F33.xx, F34.1x
Anxiety disorders	F40.xx- F42.xx, F93.0x-F93.2x, F94.0x
Adjustment disorders	F43.2x
Bipolar disorder	F30.xx-F31.xx, F34.0x
ADHD	F90.xx
Trauma disorder	F43.0x, F43.1x, F43.8x, F43.9x, F94.1x, F94.2x
Schizophrenia/psychotic disorders	F20.xx, F22.xx-F29.xx
Substance use disorder	F10-F16, F18-F19
Autism spectrum disorder (ASD)	F84.xx
Eating Disorders	F50.xx, F98.2x

© 2024 Hailu R et al. *JAMA Health Forum*.

eTable. Distribution of Visits in 2019 by Diagnostic Category Among Telemedicine-Only Clinicians vs Other Clinicians. Visit categorized by the first or primary diagnosis listed on the visit claim.

Primary Diagnosis Code on Visit	All Visits to Providers in Cohort	Visits to Telemedicine-Only Providers	Visits to Other Providers
All Visits (n)	7,947,302	1,011,398	6,935,904
Depressive disorder	29.60%	30.00%	29.60%
Anxiety disorder	21.80%	25.40%	21.30%
Adjustment disorders	17.60%	19.50%	17.40%
Bipolar disorder	10.10%	10.30%	10.10%
Attention deficit/hyperactivity disorder (ADHD)	5.30%	6.40%	5.20%
Trauma Disorder	3.90%	4.00%	3.80%
Schizophrenia/psychotic disorders	3.30%	1.50%	3.60%
Substance use disorder	2.80%	1.70%	3.00%
Autism spectrum disorder (ASD)	0.80%	0.50%	0.90%
Eating disorder	0.60%	0.70%	0.60%

© 2024 Hailu R et al. *JAMA Health Forum*.
