# Supplementary material for: Characteristics of Mental Health Specialists Who Shifted Their Practice Entirely to Telemedicine
Source: JAMA Health Forum. 2024 Jan 26;5(1):e234982. doi: 10.1001/jamahealthforum.2023.4982 (PMC10818220; doi:10.1001/jamahealthforum.2023.4982)
Supplement: Supplement 2. — Data Sharing Statement [file jamahealthforum-e234982-s002.pdf]

Data Sharing Statement

Hailu. Characteristics of Mental Health Specialists Who Shifted Their Practice Entirely to Telemedicine. *JAMA Health Forum*. Published January 26, 2024. doi:10.1001/jamahealthforum.2023.4982

Data
Data available: No

Additional Information
Explanation for why data not available: These data were accessed under a data use agreement. We'd be happy to share how we identified variables in the data.
